# *Pfiesteria* in Estuarine Waters: The Question of Health Risks

**DOI:** 10.1289/ehp.115-1849899

**Published:** 2007-03

**Authors:** Ritchie C. Shoemaker, Wayne Lawson

**Affiliations:** Center for Research on Biotoxin, Associated Illnesses, Pocomoke, Maryland, E-mail: ritchieshoemaker@msn.com; Somerset County (Maryland), Waterman’s Association (Retired), Crisfield, Maryland

The conclusion of Morris et al. (2006) that “Exposure to *Pfiesteria* Species in Estuarine Waters Is Not a Risk Factor for Illness” is unsupported because *a*) a description of *Pfiesteria*-related fish kills in the Chesapeake estuaries during 1999–2002 was omitted; *b*) quantitative data on *Pfiesteria* were not collected; *c*) data on visual contrast sensitivity (VCS) were collected but not reported; *d*) a comprehensive list of other results was not presented; and *e*) data were lost due to a 30% attrition rate. These data are needed to justify or negate the conclusion.

Since the first reports of environmental *Pfiesteria*-related illness (Shoemaker 1997) and successful treatment (Shoemaker 1998), all reports were associated with concurrent *Pfiesteria*-related fish kills (Hudnell 2005). Numerous kills were reported in the Chesapeake and North Carolina estuaries through 1998 in association with *Pfiesteria*-like zoospore concentrations of 600–35,000 cells/mL water (Glasgow 2001). Grattan et al. (1998) previously reported relationships between impairment and increased time spent in Chesapeake estuaries. Although the degree of recovery could not be determined because premorbid data were unavailable, most of the untreated participants improved within 3–6 months. However, in 1999–2002 neither Maryland nor North Carolina reported *Pfiesteria* concentrations reaching 600 cells/mL and associated fish kills (Maryland Department of Natural Resources 2006; North Carolina Department of the Environment and Natural Resources 2006). Morris et al. (2006) used a polymerase chain reaction (PCR) method to detect gene sequences supposedly specific for *Pfiesteria piscicida* and *Pfiesteria shumwayae*, although the *P. shumwayae* genus may not be *Pfiesteria* (Litaker 2005; Marshall 2006). Detections of *Pfiesteria* were rare in watermen-collected samples (0.9–2.8%). The PCR method needs only a single cell or fragment for a “hit,” and cell counts were not undertaken. There is no evidence, therefore, that *Pfiesteria* concentrations were sufficient to induce fish kills. Per a toxicology maxim, “the dose makes the poison”; a more appropriate conclusion is that exposure to estuarine *Pfiesteria* in the absence of *Pfiesteria*-related fish kills is not a risk factor for illness.

Morris et al. (2006) did not report data on VCS, the only indicator of neurologic function known to reveal deficits after recent and long-past exposures to *Pfiesteria* (Hudnell 2005). A 1997 North Carolina study assessed health risks from chronic exposures to *Pfiesteria*-inhabited estuaries (Hudnell 1998). Although most exposed cohort members reported past contact with fish kills, only two reported contact within a year. Only VCS showed a statistically significant deficit in exposed watermen relative to controls (Hudnell 1998; Hudnell et al. 2001). The VCS deficit increased with hours spent at fish kills. The 30% VCS deficit was not significantly associated with group differences in age, education, smoking, alcohol consumption, exposure to bright sunlight, or other occupational exposures. The VCS results were confirmed in studies of Chesapeake watermen (Ingsrisawang et al. 2000; Turf et al. 1999). VCS deficits of about 60% were observed in symptomatic patients within a day of fish-kill contact, and fully resolved as symptoms dissipated during cholestyramine therapy to eliminate toxins (Shoemaker 2001; Shoemaker and Hudnell 2001). Given the substantial evidence indicating that VCS is a sensitive and reliable indicator of *Pfiesteria-*associated impairment, these results are needed to draw conclusions.

Morris et al. (2006) used an extensive neuropsychological-test battery and assessed 21 symptoms. However, the results of most statistical comparisons were not presented. Global statements, such as “in no instance was there a consistent pattern of responses” (Morris et al. 2006), rather than a table of the results, left readers unable to reach conclusions independently. The authors’ statement that there were “isolated increases in RR [relative risk] for specific symptom categories” is clarified only by their example that “there was a significant increase in cognitive symptoms among exposed watermen during the active season … and postseason … in 2000” (Morris et al. 2006). Although all results need not be reported, a systematic description of primary outcomes is needed to independently reach conclusions.

Participant attrition rate was 30% (45), without explanation for 13. Six patients seeking medical care during the study period (at R.C.S.’s clinic) reported withdrawing from the study because treatment was withheld for what the patients believed to be persistent effects from long-past exposures to *Pfiesteria*-related fish kills. Each of the patients met the criteria for chronic possible estuary-associated syndrome (PEAS) following differential diagnosis, and responded positively to cholestyramine therapy. However, a description of the ultimate health outcome of people exposed to *Pfiesteria* must await follow-up results from 37 PEAS previously reported cases (Shoemaker 2001, 2006). The results of Morris et al. (2006) may be due in part to the withdrawal of participants previously affected by *Pfiesteria* exposures.

In conclusion, the results of Morris et al. (2006) are insufficient to support their conclusion that “the routine, occupational exposure to estuarine waters in which *Pfiesteria* is known to be present does not represent a significant human health risk.”
